# Should hospital-onset Adult Sepsis Event surveillance be routine… or even mandatory?

**DOI:** 10.1017/ash.2022.16

**Published:** 2022-02-28

**Authors:** Chanu Rhee, Michael Klompas

**Affiliations:** 1 Department of Population Medicine, Harvard Medical School/Harvard Pilgrim Health Care Institute, Boston, Massachusetts; 2 Division of Infectious Diseases, Department of Medicine, Brigham and Women’s Hospital, Boston, Massachusetts

## Abstract

Hospital-onset sepsis accounts for 10%–15% of all sepsis cases and is associated with very high mortality rates, yet to date most hospitals have paid little attention to tracking its incidence and outcomes. This contrasts sharply with the substantial effort that hospitals and regulatory agencies spend tracking and reporting a limited subset of healthcare-associated infections. The recent development of the Center for Disease Control and Prevention’s hospital-onset Adult Sepsis Event (ASE) definition, however, provides a validated and standardized mechanism for facilities to identify patients with nosocomial sepsis using routinely available electronic health record data. Recent data have demonstrated that hospital-onset ASE surveillance identifies many infections that are largely missed by current reportable healthcare-associated infections and that are associated with much higher mortality rates. Expanding the breadth of surveillance to include these highly consequential infections could help identify new targets for prevention and quality improvement and ultimately catalyze better outcomes for hospitalized patients. More work is needed, however, to characterize the preventability of hospital-onset ASE, develop and validate robust case-mix adjustment tools, and facilitate widespread uptake in hospitals with limited resources.

Sepsis is a leading cause of death, disability, and healthcare costs.^1–3^ Although most quality improvement efforts have focused on patients presenting to the hospital with sepsis,^4–6^ a growing body of literature highlights the high burden of hospital-onset sepsis. Between 10% and 15% of all sepsis cases are acquired in hospitals, translating into >200,000 adult cases annually in the United States.^7–9^ These patients have crude mortality rates of 20%–30%, twice as high as community-onset sepsis, and they account for >25% of all sepsis-associated deaths in US hospitals.^7,10^ Patients with hospital-onset sepsis also have higher rates of ICU admission, stay in the hospital longer, and incur 3–5-fold greater hospital costs compared to those with community-onset sepsis.^8,9^

Admittedly, much of the dismal prognosis for hospital-onset sepsis is likely attributable to its predilection for patients who are already severely ill.^10^ However, in a recent comparison of >95,000 sepsis patients drawn from 136 US hospitals, patients with hospital-onset sepsis had twice the odds of hospital death compared to patients with community-onset sepsis, even after detailed risk-adjustment.^11^ This finding suggests that differences in quality of care may also contribute to worse outcomes. Supporting this concern, a retrospective analysis of sepsis patients at 4 California hospitals reported lower bundle-compliance rates and greater delays in antibiotic therapy for those with hospital-onset sepsis.^12^

Despite these sobering statistics, no national surveillance system for hospital-onset sepsis has been established. This situation contrasts sharply with the enormous effort that hospitals and regulatory agencies spend tracking and reporting a limited subset of healthcare-associated infections (HAIs). The Centers for Medicare and Medicaid (CMS) Hospital Inpatient Quality Reporting Program, for example, requires acute-care hospitals to report central-line–associated bloodstream infections (CLABSIs), catheter-associated urinary tract infections (CAUTIs), a subset of surgical site infections (SSIs), methicillin-resistant *Staphylococcus aureus* (MRSA) bacteremia, and *Clostridioides difficile* infections to the Centers for Disease Control and Prevention (CDC) National Healthcare Safety Network (NHSN) and incorporates them into hospital payment program metrics.^13^

One important difference between these reportable HAIs and hospital-onset sepsis is that CDC has developed standardized surveillance definitions for reportable HAIs. In contrast, sepsis surveillance has traditionally been conducted using hospital discharge diagnosis codes. These are unsuitable for hospital benchmarking and quality monitoring, however, because diagnosis and coding practices for sepsis are variable and are changing over time.^14–17^ The use of administrative data to track hospital-onset sepsis is further confounded by the need to rely on the presence or absence of present-on-admission indicators, which can also be variably documented and coded.^18^

Recognizing the need for a more objective method to track sepsis incidence and outcomes, CDC created the “Adult Sepsis Event” (ASE) tool kit in 2018 to support hospitals interested in creating a robust surveillance system using routinely available electronic health record (EHR) data.^19^ The ASE definition flags patients with evidence of *presumed serious infection* (ie, collection of blood cultures and ≥4 consecutive days of antibiotics, or until 1 day prior to death, discharge to hospice, transfer to another hospital, or transition to comfort measures) plus *concurrent organ dysfunction* (ie, initiation of vasopressors, mechanical ventilation, elevated lactate, change in baseline creatinine or estimated glomerular filtration rate, increase in baseline bilirubin, or decrease in baseline platelet count) (Fig. 1). The ASE definition is based on the framework of sepsis as a suspected infection with concurrent organ dysfunction, according to the Third International Consensus Definitions for Sepsis and Septic Shock (Sepsis-3),^20^ but it has been optimized for consistent retrospective surveillance across institutions.^21^

**Fig. 1.**
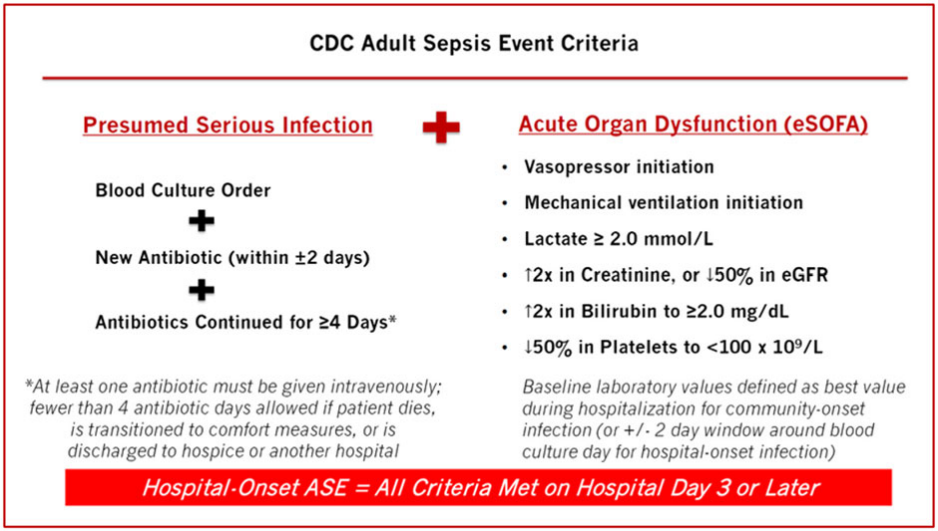
Centers for Disease Control and Prevention “Adult Sepsis Event” definition.

The ASE definition was developed by a consortium funded by the CDC Prevention Epicenters and was applied to EHR data from >400 diverse hospitals to estimate the US national burden of sepsis.^1^ The ASE was validated using >500 medical record reviews; it had better sensitivity, similar positive predictive value, and more consistent performance over time compared to sepsis diagnosis codes.^1,22^ Another important advantage over administrative data is that the ASE allows for more granular assessment of when a hospitalization sepsis event occurred.^1,14^ An ASE case is deemed hospital onset if all criteria (blood culture, first antibiotic day, and organ dysfunction) occurred on hospital day 3 or later. The hospital-onset ASE criteria performed well in an additional chart review exercise in 3 hospitals that showed very high positive predictive value for identifying nosocomial infections (as opposed to delayed presentations of present-on-admission infections). These infections had high concordance with the Sepsis-3 criteria as well as the severe sepsis time zero definition used in the CMS Severe Sepsis/Septic Shock Early Management Bundle (SEP-1) measure. ^23^

Currently, ASE surveillance is purely optional for hospitals and is not an NHSN metric. But now that this validated and potentially scalable tool is available, should hospitals begin routinely tracking hospital-onset ASEs? And should this surveillance in fact eventually become mandatory?

Several arguments support this idea. First, tracking hospital-onset ASEs provides insight into a much broader array of serious nosocomial infections beyond the select HAIs currently mandated for reporting. Illustrating this point, a retrospective analysis of 282,411 inpatients at 3 Massachusetts hospitals found that 2,301 (0.8%) met hospital-onset ASE criteria while only 1,260 (0.4%) had CMS-reportable HAIs (Fig. 2a).^23^ Furthermore, CMS-reportable HAIs were identified in only 15% of hospital-onset ASEs, and the 29% mortality rates associated with hospital-onset ASEs far exceeded the average mortality rate for the CMS-reportable HAI rate of 13% overall (Fig. 2b). Medical record reviews demonstrated that most hospital-onset ASEs were caused by nonreportable HAIs including non–ventilator-associated, hospital-acquired pneumonia, non-CLABSI bloodstream infections, and intra-abdominal infections other than *C. difficile* infection.

**Fig. 2.**
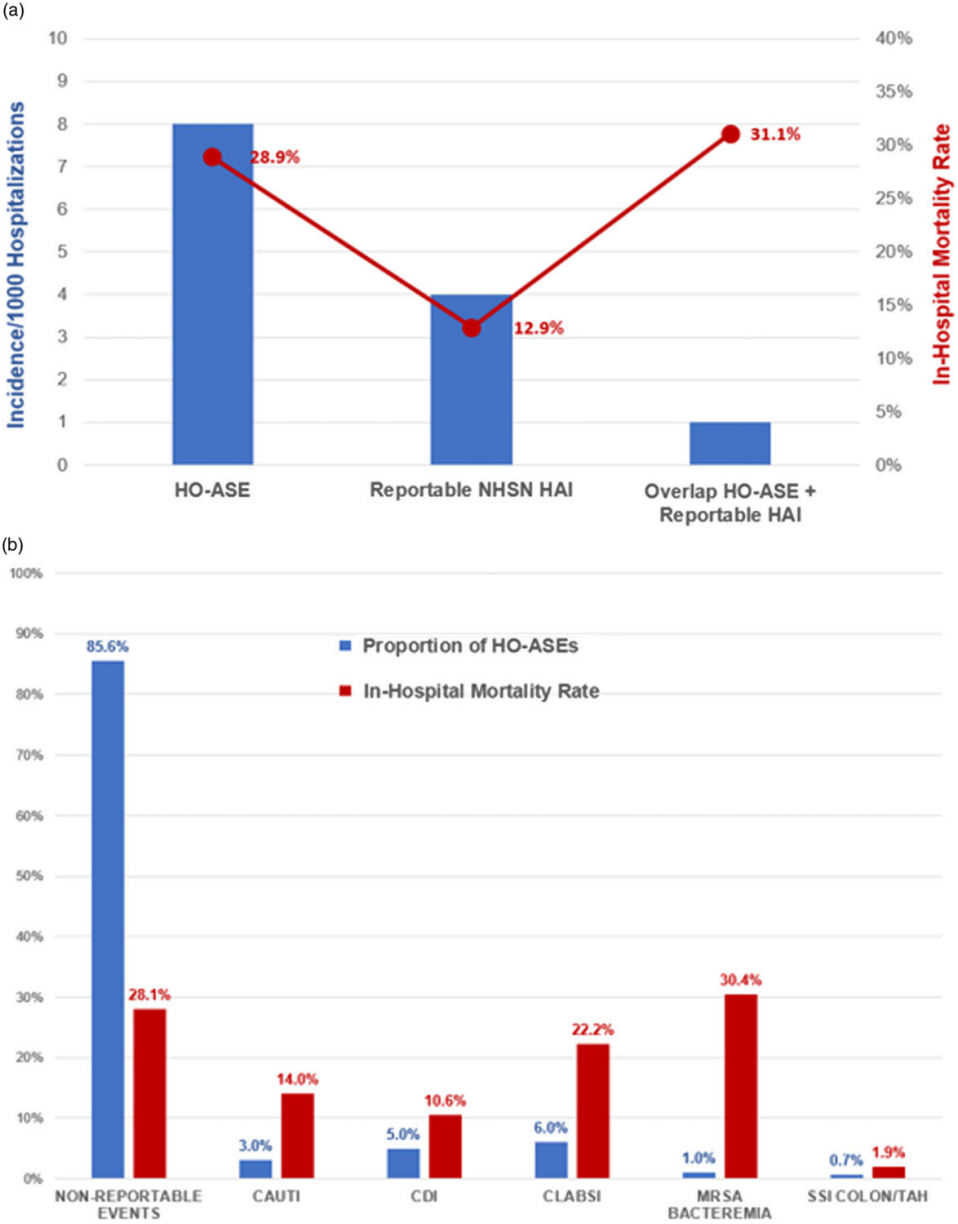
Overlap and outcomes of hospital-onset Adult Sepsis Events (HO-ASE) and reportable healthcare-associated infections (HAIs) in 3 Massachusetts hospitals. Panel A shows the overall incidence and in-hospital mortality rates of HO-ASE and 6 reportable HAIs (combined) and their overlap. Panel B shows the proportion of HO-ASEs flagged by each of the 6 reportable HAIs versus nonreportable HAIs and their associated in-hospital mortality rates. Data were obtained from 282,411 adult patients hospitalized at 3 Massachusetts hospitals between 2015 and 2018. Among them, 2,301 (0.8%) met hospital-onset ASE criteria and 1,260 (0.4%) had CMS-reportable HAIs.^23^ Note. NHSN, National Healthcare Safety Network; CAUTI, catheter-associated urinary tract infection; CDI, *Clostridioides difficile* infection; CLABSI, central-line–associated bloodstream infection; MRSA bacteremia, methicillin-resistant *Staphylococcus aureus* bacteremia; SSI Colon/TAH, surgical site infection for colon surgery or total abdominal hysterectomy.

Second, hospital-onset ASE surveillance is optimized for implementation using routinely captured EHR data. This surveillance could facilitate more efficient and objective surveillance, in contrast to some NHSN HAIs (particularly CLABSIs and SSIs) that require time-consuming and potentially subjective manual reviews.^24,25^ This change would also align with CDC’s growing movement toward objective and ideally fully automatable HAI surveillance including hospital-onset bacteremia, ventilator-associated events, and electronic definitions for non–ventilator-associated, hospital-acquired pneumonia.^26–28^

Third, surveillance of hospital-onset ASEs could help facilitate process of care reviews for sepsis arising after admission, an area that remains a large gap in current quality monitoring. Most sepsis cases currently abstracted for SEP-1 are present on admission, not only because most sepsis is community-acquired but also because the measure excludes patients receiving intravenous antibiotics for >24 hours prior to “time zero.”^29^ Reliably identifying time zero for hospital-onset sepsis cases is also much more challenging than for sepsis present on admission.^30^ Hospital-onset ASE surveillance can help flag appropriate cases for detailed review and can also narrow the approximate timeframe to review specific time-zero criteria.

To be sure, several potential limitations and knowledge gaps need to be addressed before hospital-onset ASE surveillance becomes routine. First, the preventability of hospital-onset ASEs need to be more clearly established. One advantage of the current definition of reportable HAIs is that they can be tied to specific best practices known to reduce risk (eg, aseptic central venous catheter insertion techniques for CLABSIs, diagnostic and antibiotic stewardship for *C.difficile*, etc). The breadth of conditions flagged by surveillance of hospital-onset ASEs, in contrast, may not always point to clear action items for preventing subsequent cases. Nonetheless, we believe that opening our collective eyes to the number and breadth of these severe nosocomial infections is an important step to identifying unrecognized gaps in care and catalyzing new innovations. Furthermore, hospital-onset ASE surveillance should complement, not replace, current HAI surveillance. One possible approach might be to develop a tiered system of HAI priority based on whether they also triggered hospital-onset ASE (similar to the tiered approach for superficial vs deep vs organ/space SSIs). Second, robust risk adjustment tools need to be developed for standardized hospital-onset ASE rates before any hospital benchmarking can be considered. Finally, electronic hospital-ASE surveillance requires a reasonably sophisticated EHR system and information technology expertise, and like any automated system requires periodic audits and validation. However, the CDC ASE tool kit contains detailed materials to facilitate implementation. Manual abstraction is also a suboptimal but feasible possibility for smaller facilities given that hospital-onset sepsis affects <1% of all hospitalizations.^11^

In conclusion, hospital-onset ASE surveillance provides a validated, standardized mechanism for hospitals to leverage routinely available EHR data to track many severe nosocomial infections that are largely missed by current CMS-reportable HAIs. Future work should focus on characterizing the preventability of hospital-onset ASEs, developing robust case-mix adjustment tools, and facilitating widespread uptake in hospitals with limited resources. Ultimately, we believe that routine—and even mandatory—reporting of hospital-ASEs could help elucidate the full spectrum of serious nosocomial infections, identify new targets for prevention and quality improvement, and catalyze better outcomes for hospitalized patients.

## References

[1] Rhee C , Dantes R , Epstein L , et al. Incidence and trends of sepsis in US hospitals using clinical vs claims data, 2009–2014. JAMA 2017;318:1241–1249. 28903154 10.1001/jama.2017.13836 PMC5710396

[2] Rudd KE , Johnson SC , Agesa KM , et al. Global, regional, and national sepsis incidence and mortality, 1990–2017: analysis for the Global Burden of Disease Study. Lancet 2020;395:200–211. 31954465 10.1016/S0140-6736(19)32989-7 PMC6970225

[3] Buchman TG , Simpson SQ , Sciarretta KL , et al. Sepsis among Medicare beneficiaries: 1. the burdens of sepsis, 2012–2018. Crit Care Med 2020;48:276–288. 32058366 10.1097/CCM.0000000000004224 PMC7017943

[4] Raghavan M , Marik PE. Management of sepsis during the early “golden hours.” J Emerg Med 2006;31:185–199. 17044583 10.1016/j.jemermed.2006.05.008

[5] Seymour CW , Gesten F , Prescott HC , et al. Time to treatment and mortality during mandated emergency care for sepsis. N Engl J Med 2017;376:2235–2244. 28528569 10.1056/NEJMoa1703058 PMC5538258

[6] Investigators P , Rowan KM , Angus DC , et al. Early, goal-directed therapy for septic shock—a patient-level meta-analysis. N Engl J Med 2017;376:2223–2234. 28320242 10.1056/NEJMoa1701380

[7] Rhee C , Wang R , Zhang Z , et al. Epidemiology of hospital-onset versus community-onset sepsis in US hospitals and association with mortality: a retrospective analysis using electronic clinical Data. Crit Care Med 2019;47:1169–1176. 31135503 10.1097/CCM.0000000000003817 PMC6697188

[8] Page DB , Donnelly JP , Wang HE. Community-, healthcare-, and hospital-acquired severe sepsis hospitalizations in the university health system consortium. Crit Care Med 2015;43:1945–1951. 26110490 10.1097/CCM.0000000000001164 PMC4537676

[9] Paoli CJ , Reynolds MA , Sinha M , Gitlin M , Crouser E. Epidemiology and costs of sepsis in the United States—an analysis based on timing of diagnosis and severity level. Crit Care Med 2018;46:1889–1897. 30048332 10.1097/CCM.0000000000003342 PMC6250243

[10] Rhee C , Jones TM , Hamad Y , et al. Prevalence, underlying causes, and preventability of sepsis-associated mortality in US acute-care hospitals. JAMA Netw Open 2019;2:e187571. 30768188 10.1001/jamanetworkopen.2018.7571 PMC6484603

[11] Rhee C , Wang R , Zhang Z , et al. Epidemiology of hospital-onset versus community-onset sepsis in US hospitals and association with mortality: a retrospective analysis using electronic clinical data. Crit Care Med 2019;47:1169–1176. 31135503 10.1097/CCM.0000000000003817 PMC6697188

[12] Baghdadi JD , Wong MD , Uslan DZ , et al. Adherence to the SEP-1 sepsis bundle in hospital-onset versus community-onset sepsis: a multicenter retrospective cohort study. J Gen Intern Med 2020;35:1153–1160. 32040837 10.1007/s11606-020-05653-0 PMC7174506

[13] Hsu HE , Wang R , Broadwell C , et al. Association between federal value-based incentive programs and healthcare-associated infection rates in safety-net and non–safety-net hospitals. JAMA Netw Open 2020;3:e209700. 32639568 10.1001/jamanetworkopen.2020.9700 PMC7344380

[14] Rhee C , Kadri SS , Danner RL , et al. Diagnosing sepsis is subjective and highly variable: a survey of intensivists using case vignettes. Crit Care 2016;20:89. 27048508 10.1186/s13054-016-1266-9 PMC4822273

[15] Tang KL , Lucyk K , Quan H. Coder perspectives on physician-related barriers to producing high-quality administrative data: a qualitative study. CMAJ Open 2017;5:E617–E622. 10.9778/cmajo.20170036 PMC5621953 28827414

[16] Rhee C , Jentzsch MS , Kadri SS , et al. Variation in identifying sepsis and organ dysfunction using administrative versus electronic clinical data and impact on hospital outcome comparisons. Crit Care Med 2019;47:493–500. 30431493 10.1097/CCM.0000000000003554 PMC7970408

[17] Rhee C , Klompas M. Sepsis trends: increasing incidence and decreasing mortality, or changing denominator? J Thorac Dis 2020;12:S89–S100. 32148931 10.21037/jtd.2019.12.51 PMC7024753

[18] Goldman LE , Chu PW , Osmond D , Bindman A. The accuracy of present-on-admission reporting in administrative data. Health Serv Res 2011;46:1946–1962. 22092023 10.1111/j.1475-6773.2011.01300.x PMC3393034

[19] Hospital toolkit for adult sepsis surveillance. Centers for Disease Control and Prevention website. https://www.cdc.gov/sepsis/pdfs/Sepsis-Surveillance-Toolkit-Mar-2018_2508.pdf. Published 2018. Accessed February 4, 2022.

[20] Singer M , Deutschman CS , Seymour CW , et al. The Third International Consensus Definitions for Sepsis and Septic Shock (Sepsis-3). JAMA 2016;315:801–810. 26903338 10.1001/jama.2016.0287 PMC4968574

[21] Rhee C , Dantes RB , Epstein L , Klompas M. Using objective clinical data to track progress on preventing and treating sepsis: CDC’s new ‘adult sepsis event’ surveillance strategy. BMJ Qual Saf 2019;28:305–309. 10.1136/bmjqs-2018-008331 PMC6557151 30254095

[22] Rhee C , Kadri S , Huang SS , Murphy MV , Li L , Platt R , Klompas M. Objective sepsis surveillance using electronic clinical data. Infect Control Hosp Epidemiol. 2016;37(2):163–171. 26526737 10.1017/ice.2015.264 PMC4743875

[23] Page B , Klompas M , Chan C , et al. Surveillance for healthcare-associated infections: hospital-onset adult sepsis events versus current reportable conditions. Clin Infect Dis 2021;73:1013–1019. 33780544 10.1093/cid/ciab217

[24] Mayer J , Greene T , Howell J , et al. Agreement in classifying bloodstream infections among multiple reviewers conducting surveillance. Clin Infect Dis 2012;55:364–370. 22539665 10.1093/cid/cis410

[25] Keller SC , Linkin DR , Fishman NO , Lautenbach E. Variations in identification of healthcare-associated infections. Infect Control Hosp Epidemiol 2013;34:678–686. 23739071 10.1086/670999 PMC3981741

[26] Magill SS , Klompas M , Balk R , et al. Developing a new, national approach to surveillance for ventilator-associated events: executive summary. Infect Control Hosp Epidemiol 2013;34:1239–1243. 24225607 10.1086/673463

[27] Ji W , McKenna C , Ochoa A , et al. Development and assessment of objective surveillance definitions for nonventilator hospital-acquired pneumonia. JAMA Netw Open 2019;2:e1913674. 31626321 10.1001/jamanetworkopen.2019.13674 PMC6813588

[28] Dantes RB , Rock C , Milstone AM , et al. Preventability of hospital onset bacteremia and fungemia: a pilot study of a potential healthcare-associated infection outcome measure. Infect Control Hosp Epidemiol 2019;40:358–361. 30773166 10.1017/ice.2018.339 PMC10848935

[29] Baghdadi JD , Brook RH , Uslan DZ , et al. Association of a care bundle for early sepsis management with mortality among patients with hospital-onset or community-onset sepsis. JAMA Intern Med 2020;180:707–716. 32250412 10.1001/jamainternmed.2020.0183 PMC7136852

[30] Rhee C , Brown SR , Jones TM , et al. Variability in determining sepsis time zero and bundle compliance rates for the centers for medicare and medicaid services SEP-1 measure. *Infect. Control Hosp. Epidemiol* 2018. 10.1017/ice.2018.134 PMC7977505 29932042

